# Accumulation of Self-Reactive Naïve and Memory B Cell Reveals Sequential Defects in B Cell Tolerance Checkpoints in Sjögren’s Syndrome

**DOI:** 10.1371/journal.pone.0114575

**Published:** 2014-12-23

**Authors:** Elisa Corsiero, Nurhan Sutcliffe, Costantino Pitzalis, Michele Bombardieri

**Affiliations:** Centre for Experimental Medicine & Rheumatology, William Harvey Research Institute, Barts and The London School of Medicine & Dentistry, Queen Mary University of London, John Vane Science Centre, Charterhouse Square, London EC1M 6BQ, United Kingdom; University of Nebraska-Lincoln, United States of America

## Abstract

Sjögren’s syndrome (SS) is an autoimmune disease characterised by breach of self-tolerance towards nuclear antigens resulting in high affinity circulating autoantibodies. Although peripheral B cell disturbances have been described in SS, with predominance of naïve and reduction of memory B cells, the stage at which errors in B cell tolerance checkpoints accumulate in SS is unknown. Here we determined the frequency of self- and poly-reactive B cells in the circulating naïve and memory compartment of SS patients. Single CD27−IgD+ naïve, CD27+IgD+ memory unswitched and CD27+IgD− memory switched B cells were sorted by FACS from the peripheral blood of 7 SS patients. To detect the frequency of polyreactive and autoreactive clones, paired Ig V_H_ and V_L_ genes were amplified, cloned and expressed as recombinant monoclonal antibodies (rmAbs) displaying identical specificity of the original B cells. IgV_H_ and V_L_ gene usage and immunoreactivity of SS rmAbs were compared with those obtained from healthy donors (HD). From a total of 353 V_H_ and 293 V_L_ individual sequences, we obtained 114 rmAbs from circulating naïve (n = 66) and memory (n = 48) B cells of SS patients. Analysis of the Ig V gene repertoire did not show significant differences in SS vs. HD B cells. In SS patients, circulating naïve B cells (with germline V_H_ and V_L_ genes) displayed a significant accumulation of clones autoreactive against Hep-2 cells compared to HD (43.1% vs. 25%). Moreover, we demonstrated a progressive increase in the frequency of circulating anti-nuclear naïve (9.3%), memory unswitched (22.2%) and memory switched (27.3%) B cells in SS patients. Overall, these data provide novel evidence supporting the existence of both early and late defects in B cell tolerance checkpoints in patients with SS resulting in the accumulation of autoreactive naïve and memory B cells.

## Introduction

Sjögren’s syndrome (SS) is a chronic inflammatory/autoimmune disease characterised by immune cell infiltration in the salivary and lacrimal glands leading to the classical signs and symptoms of xerostomia (dry mouth) and keratoconjuctivitis (dry eyes) sicca [1]. Together with exocrine dysfunction, the hallmark of SS is the presence of circulating autoantibodies directed against organ- and non-organ-specific autoantigens. Sera of 90% of SS patients are characterised by the presence of antinuclear antibodies (ANA), most of which react against the ribonucleoproteins Ro/SSA and/or La/SSB [2]. In addition, several other autoantibody specificities, including those against alpha-fodrin, carbonic anhydrase II and the muscarinic acetylcholine receptor 3 (M3R) have been described in SS patients and suggested to be involved in salivary dysfunction, especially the latter [1], [3]–[6].

Besides the presence of autoantibodies, SS patients are characterised by profound disturbances in the frequency of different B cell subpopulations, both in the peripheral compartment and in the inflamed salivary glands. Typically, SS patients show a large predominance of circulating CD27− naïve B cells and a significant reduction of peripheral CD27+ memory B cells, in particular the memory unswitched CD27+IgD+ subpopulation [7]. Conversely, a significant accumulation of both CD27+ memory and (to a lesser extent) CD27− naïve B cells have been described in the SS salivary glands [7]–[9], possibly as a result of increased migration/retention in the inflamed tissue, particularly in the context of ectopic lymphoid structures which develop in ∼30% of SS salivary glands [10]–[12].

However, despite the evidence of profound peripheral and lesional B cell disturbances and humoral autoimmunity, the stage of B cell development at which the breach of self-tolerance and the onset of B cell autoreactivity develop in SS patients is still unclear.

In physiological conditions, self-reactive (and polyreactive) B cells, which are normally generated in the bone marrow as a consequence of random V(D)J recombination process, are silenced before entering the mature peripheral B cell compartments at two major tolerance checkpoints. The first occurs in the bone marrow between the early immature and immature B cell stage, while the second checkpoint between the transitional and the mature naïve B cell stage allowing the reduction of autoreactive/polyreactive B cells from the peripheral, circulating naïve pool [13]–[15]. Additionally, a third self-tolerance checkpoint ensures the removal of most poly- and self-reactive antibodies during the IgM+ memory B cell differentiation (pre-GC checkpoint) [13]–[15]. Conversely, during autoimmune diseases, such as rheumatoid arthritis (RA) and systemic lupus erythematosus (SLE), perturbation of these early B cell tolerance checkpoints have been described, as demonstrated by the increased frequency of polyreactive and self-reactive B cells in the naïve and memory peripheral B cell compartment [13], [16].

However, whether similar defects in B cell differentiation tolerance checkpoint are present in SS patients is currently unknown. In order to address this aspect, in this work we characterised the frequency of self- and poly-reactive circulating naïve, unswitched memory and switched memory B cells from patients with SS at the single cell level through the cloning and *in vitro* expression of complete (i.e., IgH+IgL chains) rmAbs which bear identical specificity to the original B cells [14], [17].

## Materials and Methods

### Patients and controls

Peripheral blood mononuclear cells (PBMC) were obtained from 12 patients with a diagnosis of primary/secondary SS according to the American-European Consensus Group classification criteria after informed consent (National Research Ethics Service Committee London - LREC 05/Q0707/1. This board specifically approved the collection of peripheral blood from patients with primary Sjögren’s syndrome, including the isolation of peripheral blood mononuclear cells for downstream analysis) [18] (Table 1). Written consent was obtained from all participants using a patient information sheet (PIS) and consent form approved by the ethics committee stated above. Control PBMCs samples were obtained from 7 HD including fresh naïve B cells obtained at QMUL (SG and HJ) as well as previously characterised Ig V_H_ and V_L_ plasmids and/or Ig V_H_ and V_L_ sequences and autoreactive profiles obtained from IgD+ naïve [19], IgM+ memory B cells [20] and IgG+ memory B cells [21] kindly provided by Prof Hedda Wardemann at Max Planck Institute for Infection Biology (Berlin, Germany).

**Table 1:** SS patients analysed in this study.

SS patients	SS1	SS2	SS3	SS4	SS5	SS6^a^	SS8	SS9	SS10	SS11	SS12	SS13
**Age**	64	64	59	54	39	59	68	64	48	62	41	56
**Gender**	F	F	F	F	F	F	F	F	F	M	F	F
**Age SS onset**	52	50	43	44	32	43	66	62	43	53	26	46
**Disease duration (Years)**	12	14	16	10	7	16	2	2	5	9	14	10
**Diagnosis**	pSS^b^	pSS	pSS	pSS	pSS	pSS	pSS	pSS	sSS^b^/RA^b^	pSS	pSS	pSS
**ANA**	+	+	+	+	+	+	+	+	+	+	+	+
**Anti-Ro/SSA**	+	+	+	+	+	+	−	−	+	+	+	+
**Anti-La/SSB**	+	−	−	+	−	−	−	−	−	+	+	+
**Treatment**	HCQ^c^	HCQ	HCQ	HCQ	N^c^	N	N	HCQ	HCQ MTX^c^	HCQ	HCQ PDN^c^	HCQ PDN

Demographic and clinical characteristics of the 12 patients with Sjögren’s syndrome (SS).

a Patient SS7 is the same as patient SS13 but analysed at different time points.

b pSS = primary Sjögren’s syndrome; sSS = secondary Sjögren’s syndrome; RA = rheumatoid arthritis.

c HCQ = Hydroxychloroquine; MTX = Methotrexate; PDN = prednisolone; N = no treatment.

### Phenotypic characterization of circulating B cells in SS patients

PBMC were obtained from heparinized blood by centrifugation on Ficoll-Paque gradients. Blood was diluted with 1X PBS, supplemented with 2.5 mM EDTA and loaded over a Ficoll-Paque. Density gradient centrifugation was performed at 2000 rpm at room temperature for 25 min to separate mononuclear cells. Mononuclear cells at the interface containing PBMC were collected and washed twice with PBS. PBMC viability was determined by Trypan blue exclusion test. Immunofluorescence labeling for flow cytometry was performed by staining the purified mononuclear cells on ice with PerCP-Cy5.5 anti-human CD19 (clone SJ25C1; BD Biosciences), APC anti-human CD27 (clone O323; eBioscience), PE anti-human IgD (clone IA6-2; BD Biosciences), and FITC anti-human CD3 (clone HIT3a, eBioscience) in order to differentiate CD3−CD19+CD27−IgD+ naïve B cells, CD3−CD19+CD27+IgD+ unswitched memory B cells and CD3−CD19+CD27+IgD− switched memory B cells as previously described [7]. Flow cytometric analysis was performed with a FACSAria flow cytometer (Becton Dickinson); 50,000 events were collected for each analysis.

### Isolation of single human B cells by fluorescence activated cell sorting

**Figure 1:**
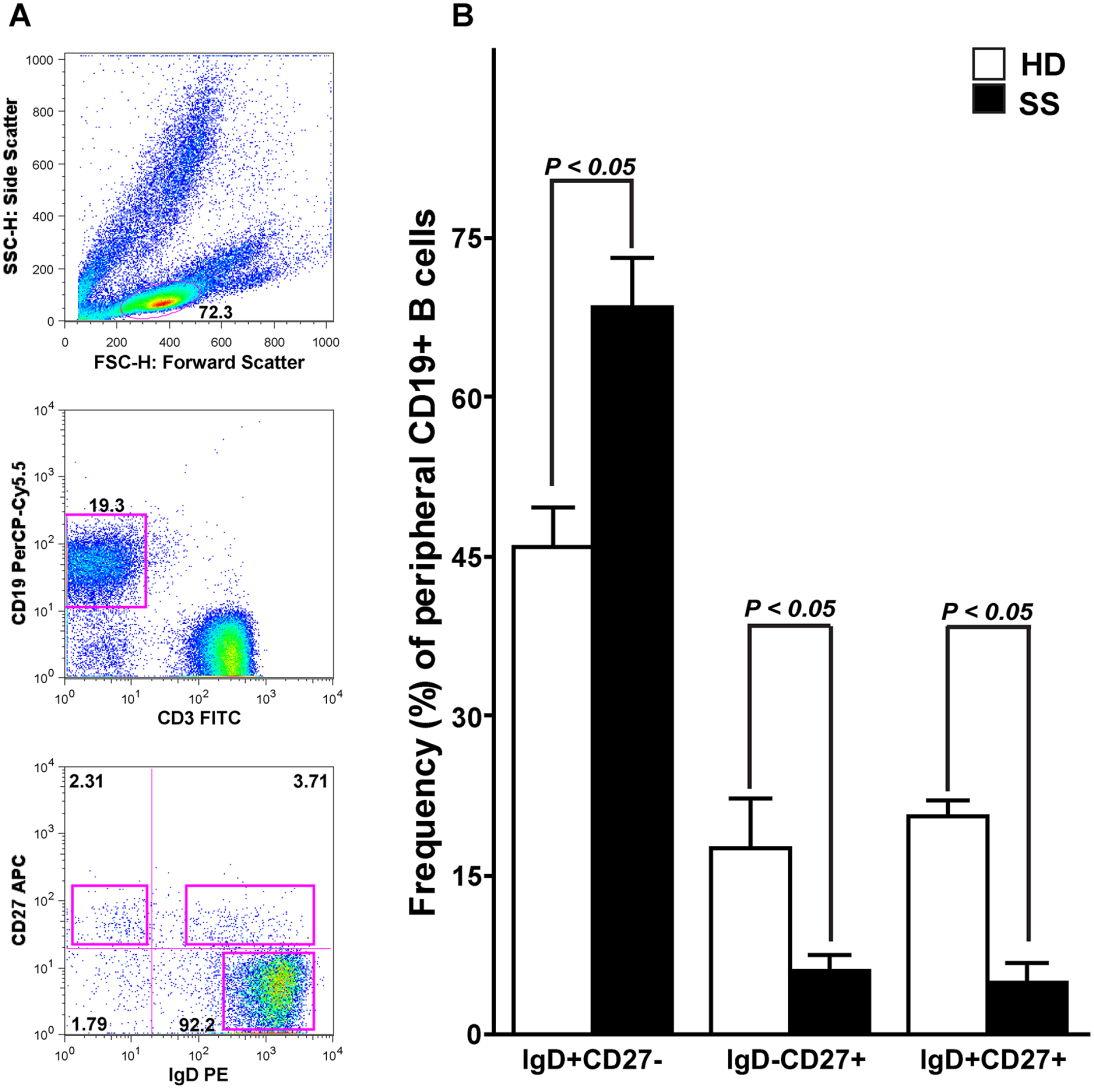
Isolation strategy of single naïve B cells and comparison of the frequencies of naïve, memory switched and unswitched B cells in SS patients and HD. (**A**) PBMC from SS patients were surface labeled with fluorochrome-coupled anti-CD19, anti-CD3, anti-IgD and anti-CD27. CD19+CD3− cells were gated and analyzed for IgD and CD27 expression. (**B**) The frequencies of peripheral naïve (CD3−CD19+CD27−IgD+), memory switched (CD3−CD19+CD27+IgD–) and unswitched (CD3−CD19+CD27+IgD+) B cells among all CD19+ B cells are shown. Differences between patients and HD were statistically significant using the nonparametric Mann-Whitney U test (*p* value is reported over each graph). Error bars indicate standard error of the mean (SEM) for individual patient or control.

PBMC from 4 out of 12 SS patients (SS3, SS5, SS12, and SS13) were used for the isolation of single CD3−CD19+CD27−IgD+ naïve B cells. For the isolation of single CD3−CD19+CD27+IgD+ unswitched memory we used PBMC from 6 out of 12 SS patients (SS3, SS4, SS5, SS6, SS12, and SS13) while PBMC from 7 out of 12 SS patients (SS3, SS4, SS5, SS6, SS9, SS12, and SS13) were used for the isolation of CD3−CD19+CD27+IgD− switched memory B cells. The gating and sorting strategy is described in Fig. 1A. Single cells were sorted on a FACSAria (Becton Dickinson) directly into 96-well plates (Eppendorf) containing 4 µl/well of ice-cold 0.5X PBS containing 100 mM DTT (Invitrogen), 40 U/µl RNasin Ribonuclease Inhibitor (Promega) as previously described [17]. Plates were sealed with adhesive PCR foil (4titude) and immediately frozen on dry ice before storage at −80°C.

### Single cell RT-PCR and immunoglobulin V gene amplification

cDNA was synthesized in a total volume of 14.5 µl per well in the original 96-well sorting plate as previously described [17]. In brief, total RNA from single cells was reverse transcribed in nuclease-free water (Qiagen) using 300 ng/µl random hexamer primers (Roche), 25 mM each nucleotide dNTP-mix (Invitrogen), 100 mM DTT (Invitrogen), 10% NP-40 (Sigma), 40 U/µl RNasin (Promega), and 50 U Superscript III reverse transcriptase (Invitrogen). Reverse transcription, single-cell RT-PCR reactions, and immunoglobulin V gene amplification were performed as described [17] with the exception of the use of a novel Cµ internal primer (GGGAATTCTCACAGGAGACGA) in the second round of the nested PCR. Briefly, for each cell IgH and corresponding IgL chain (Igκ and Igλ) gene transcripts were amplified independently by nested PCR starting from 3 µl of cDNA as template. All PCR reactions were performed in 96-well plates in a total volume of 40 µl per well containing 50 mM each primer [17], 25 mM each nucleotide dNTP-mix (Invitrogen) and 1.2 U HotStar Taq DNA polymerase (Qiagen). All nested PCR reactions with family-specific primers were performed with 3 µl of unpurified first PCR product. The complete sequence of primers used in this work is reported in S1 Table.

### Ig gene sequence analysis

Aliquots of V_H_, V_κ_ and V_λ_ chain second PCR products were sequenced with the respective reverse primer (Beckman Coulter Genomics) and the sequences were analyzed by IgBlast (http://www.ncbi.nlm.nih.gov/igblast/) to identify germline V(D)J gene segments with highest homology. IgH complementary determining region (CDR)3 length and the number of positively (Histidine(H), Arginine (R), Lysine (K)) and negatively charged (Aspartate (D), Glutamate (E)) amino acids were determined as previously described [14], [17]. Out of 96 single naïve B cell sorted from each of the 4 SS patients, we obtained successful sequences from a total of 119 different V_H_ and J_H_ regions (SS3 = 19, SS5 = 53, SS12 = 17, SS13 = 30), 73 V_κ_ and J_κ_ regions (SS3 = 17, SS5 = 31, SS12 = 2, SS13 = 23), and 36 V_λ_ and J_λ_ regions (SS3 = 13, SS5 = 10, SS12 = 10, SS13 = 3) (S2 Table). For the memory unswitched B cells, we obtained successful sequences from a total of 131 different V_H_ and J_H_ regions (SS3 = 33, SS4 = 18, SS5 = 11, SS6 = 18, SS12 = 26, SS13 = 25), 74 V_κ_ and J_κ_ regions (SS3 = 23, SS4 = 8, SS5 = 8, SS6 = 11, SS12 = 10, SS13 = 14), and 37 V_λ_ and J_λ_ regions (SS3 = 8, SS4 = 6, SS5 = 2, SS6 = 5, SS12 = 5, SS13 = 11) (S4 Table). Finally, for the memory switched B cells, we obtained successful sequences from a total of 103 V_H_ and J_H_ regions (SS3 = 24, SS4 = 21, SS5 = 8, SS6 = 6, SS9 = 20, SS12 = 19, SS13 = 5), 41 V_κ_ and J_κ_ regions (SS3 = 14, SS4 = 3, SS5 = 7, SS6 = 4, SS9 = 3, SS12 = 9, SS13 = 1), and 32 V_λ_ and J_λ_ regions (SS3 = 10, SS4 = 7, SS5 = 0, SS6 = 2, SS9 = 4, SS12 = 4, SS13 = 5) (S5 Table). V_H_, V_κ_ and V_λ_ sequences from SS patients were compared with V_H_, V_κ_ and V_λ_ sequences from either freshly obtained IgD+ naïve B cells (S3 Table) or from a re-analysis of sequences from memory unswitched and memory switched B cells from HD previously published [20], [21] and kindly provided by Prof Hedda Wardemann at Max Planck Institute for Infection Biology (Berlin, Germany).

Clones with matching productive V_H_ and V_L_ products were used for downstream cloning and recombinant antibody expression. All productive V_H_ and V_L_ products from naïve B cells displayed unmutated germ line sequences (data not shown).

Immunoglobulin Analysis Tool (IgAT) software was used to calculate the probability of antigen-driven selection within the Ig repertoire of memory unswitched and switched B cells [22]. IgAT uses the algorithms generated by Chang and Casali [23] and by Lossos et al. [24] in order to identify sequences that are indicative of an antigen-driven selection.

### Expression vector cloning and monoclonal antibody production

The expression vector cloning strategy and antibody production were performed as previously described [17]. Briefly, before cloning all PCR products were digested with the respective restriction enzymes AgeI, Sall, BsiWI and XhoI (all from NEB). Digested PCR products were ligated using the T4 DNA Ligase (NEB) into human IgG1, Igκ or Igλ expression vector. Competent *E. coli* DH5α bacteria (Invitrogen) were transformed at 42°C with 3 µl of the ligation product. Colonies were screened by PCR and PCR products of the expected size (650 bp for Igγ1, 700 bp for Igκ and 590 bp for Igλ) were sequenced to confirm identity with the original PCR products.

To express the antibodies *in vitro*, cells of the Human Embryonic Kidney (HEK) 293T cell line were cultured in 6 well plates (Falcon, BD) and co-transfected with plasmids encoding the IgH and IgL chain originally amplified from the same B cell. Transient transfection of exponentially growing 293T cells was performed by Polyethylenimine (Sigma) at 60–70% cell confluency. Tissue culture supernatants with the secreted antibodies were stored at 4°C with 0.05% sodium azide. Recombinant antibody concentrations were determined by IgG ELISA before and after purification with Protein G beads (GE Healthcare).

### Characterization of polyreactivity and self-reactivity by ELISAs and Indirect Immunofluorescence Assay (IFA)

To test the reactivity against different allo- and auto-antigens, first supernatants were tested for polyreactivity against double and single-stranded DNA (dsDNA and ssDNA), lipopolysaccharide (LPS) and insulin by ELISA as previously reported [17]. Antibodies that reacted against at least two structurally diverse self- and non-self-antigens were defined as polyreactive [14], [17]. Internal controls for polyreactivity were kindly provided by Prof Hedda Wardemann at Max Planck Institute for Infection Biology (Berlin, Germany) and added on each plate consisting of the rmAbs mGO53 (negative), JB40 (low polyreactive), and ED38 (highly polyreactive) as previously reported [17]. Second, to analyse the autoreactivity profile of the cloned antibodies, purified IgG were screened for self-reactivity against Hep-2 cells using the indirect-immunofluorescence assay (IFA, Orgentec).

For the polyreactivity ELISAs, antibodies were tested at 1 µg/ml and the cut-off optical density (OD) at which all antibodies were considered reactive was determined for each experiment based on the mean OD plus 2 standard deviations of the mGO53 control antibody as previously described [14], [17]. Finally, for detection of ANA using Hep-2 coated slides as substrate, purified antibodies were incubated at 10 µg/ml. Alexa Fluor 488-conjugated goat anti-human IgG (Invitrogen) was used as detection antibody. Hep-2 staining patterns were visualised using an Olympus BX60 microscope and digital images acquired using identical exposure times throughout (1000 msec). ANA were scored independently by 3 trained observers (EC, MB and SG, see acknowledgements) and considered positive in case of concordance by at least 2 observers. Antibodies expressed at a concentration below 1 µg/ml were excluded from the analysis.

### Statistical analysis

Differences in quantitative variables were analyzed by the Mann-Whitney U test when comparing two groups and by the Kruskal-Wallis with Dunn’s post-test when comparing multiple groups. χ^2^ test with Yates’ correction when required or Fisher’s exact test when appropriate were used to evaluate associations of qualitative variables in the different groups. All the statistical analyses were performed using GraphPad Prism version 5.01 for Windows (GraphPad, San Diego, CA). A *p* value <0.05 was considered statistically significant.

## Results

### Disturbances in peripheral B cell subpopulation in SS patients

Previous studies analyzing B cell subpopulations in SS patients have shown a significantly reduction of circulating CD27+ memory B cells with accumulation of CD27− naïve B cells [7]. Thus, we first used 4-color flow cytometry to analyze the frequency of naïve (CD3−CD19+CD27−IgD+), class-switched memory B cells (CD3−CD19+CD27+IgD–) and unswitched memory B cells (CD3−CD19+CD27+IgD+) in patients with SS (Fig. 1A). This analysis confirmed that in our cohort of SS patients the frequency of circulating CD27− naïve B cells was increased compared to HD (mean±SD 68.5±16.0% versus 45.8±6.7%, respectively), whereas the frequency of CD27+ switched (6.0±5.2% versus 17.5±8.1%) and unswitched (4.9±6.6% versus 20.6±2.4%) memory B cells was significantly reduced (Fig. 1B, including *p* values).

### Naïve B cell Ig V_H_ and V_L_ gene repertoire analysis

**Figure 2:**
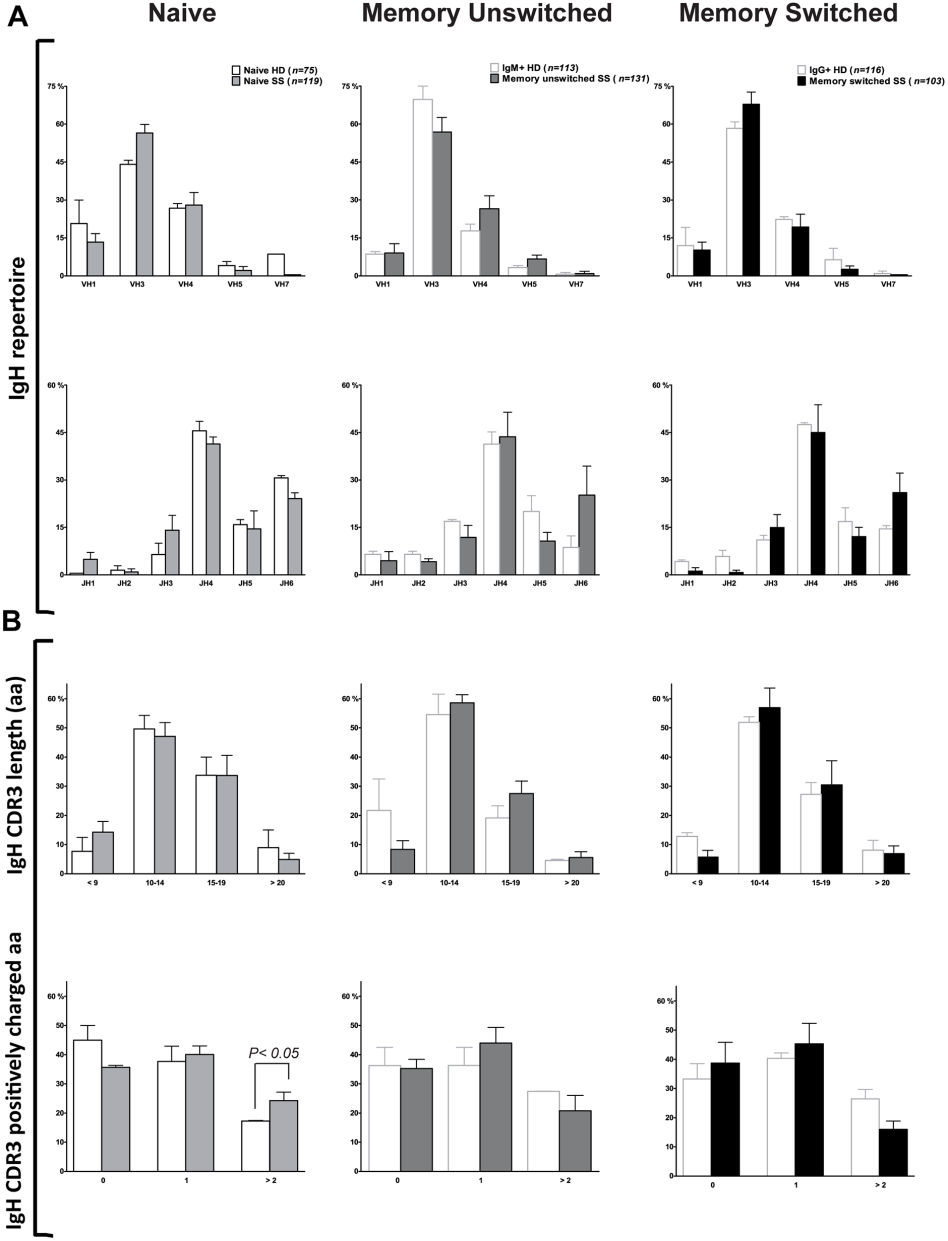
Ig gene analysis of naïve and memory B cells from SS patients and HD. Single naïve and memory unswitched and switched B cell antibodies from SS patients and HD [19]–[21] were analyzed for (**A**) Ig V_H_ family and J_H_ gene usage, and (**B**) IgH CDR3 aa length and number of positive charges. The absolute number of sequences analyzed is reported over the graph. Error bars in bar graphs indicate standard error of mean (SEM) for individual patient or control.

**Figure 3:**
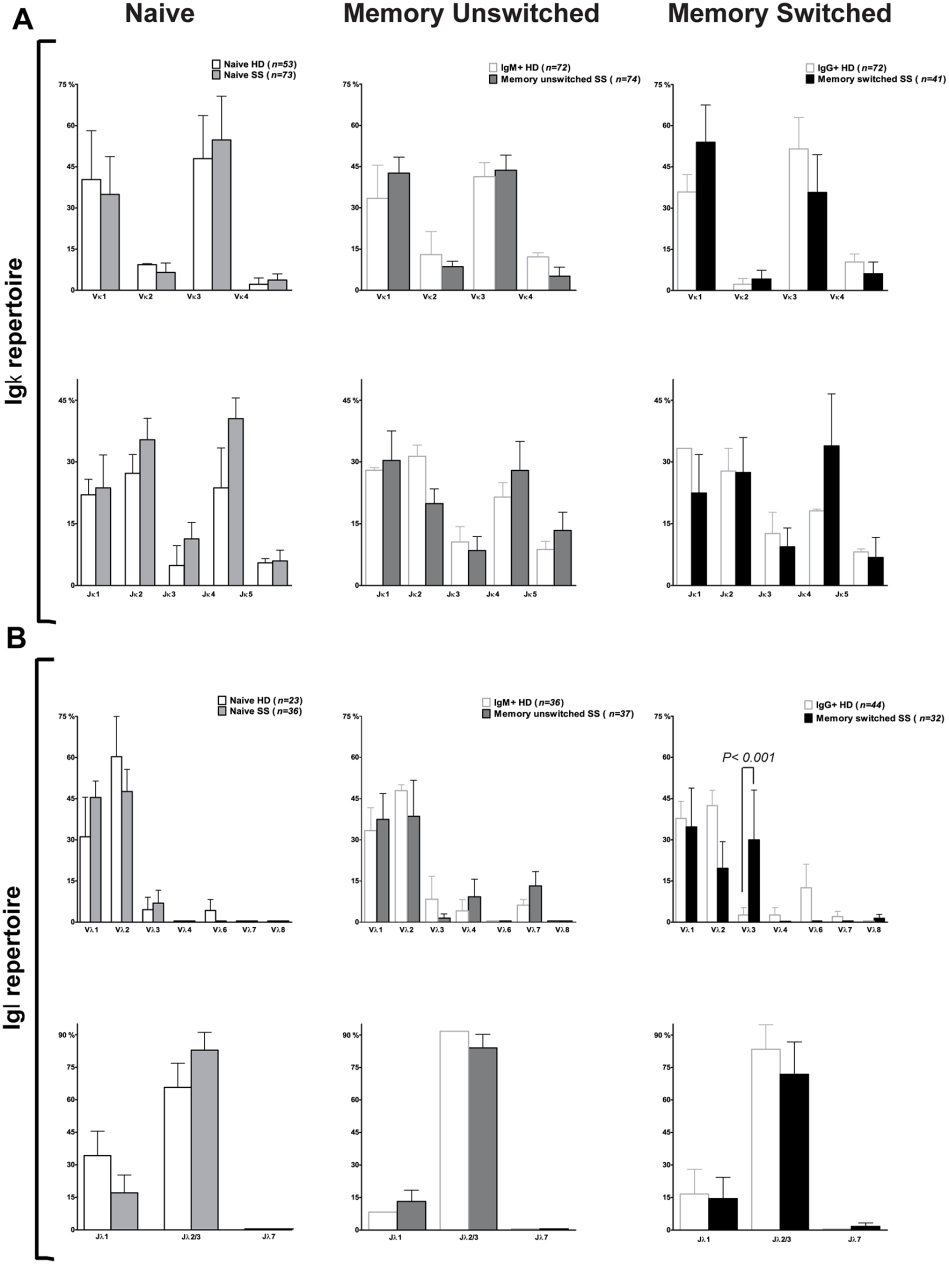
Ig gene analysis of naïve and memory B cells from SS patients and HD. Single naïve and memory unswitched and switched B cell antibodies from SS patients and HD [19]–[21] were analyzed for Ig V_κ_ and J_κ_ (**A**) and Ig V_λ_ and J_λ_ (**B**) gene usage. The absolute number of sequences analyzed is reported over each graph. Error bars in bar graphs indicate standard error of mean (SEM) for individual patient or control.

To characterise the Ig genes expressed by naïve B cells in SS patients, we sorted CD3−CD19+CD27−IgD+ naïve B cells as single cells by flow cytometry from peripheral blood. The analysis of 119 V(D)J gene segments demonstrated a similar variable heavy (V_H_), diversity (D) and joining (J)_H_ gene repertoire in control and SS naïve B cells (Fig. 2A). As expected, V_H_3 was expressed more frequently, followed by V_H_4 and V_H_1 both in SS patients and HD. We found a frequent usage of J_H_4 followed by J_H_6, J_H_5 and J_H_3 while J_H_1 and J_H_2 were expressed less frequently both in SS patients and controls, as previously shown [25]. Similarly, no significant differences in V and J gene usage for kappa (κ, 73 sequences) and lambda (λ, 36 sequences) chains were observed between SS patients and HD (Figs. 3A and 3B). SS naïve Ig gene transcripts displayed a significantly higher frequency of positively charged IgH complementary determining regions 3 (CDR3), a feature commonly associated with autoreactive antibodies [14] (Fig. 2B, bottom panel). However, the frequency of long CDR3, another feature frequently displayed by polyreactive antibodies [14], was similar between HD and SS patients (Fig. 2B, top panel). Thus, we concluded that overall there are no major abnormalities in the Ig gene repertoire of circulating naïve B cells in SS patients as previously reported [7].

### Memory B cell Ig H and Ig L V gene repertoire and mutational analysis

CD3−CD19+IgD−CD27+ memory switched and CD3−CD19+IgD+CD27+ memory unswitched B cells were sorted as single cell by flow cytometry from peripheral blood of SS patients. The analysis of 103 V(D)J gene segments from the memory switched B cells and 131 V(D)J gene segments from the memory unswitched B cells demonstrated a similar V_H_, D and J_H_ gene repertoire in both memory B cell compartments (Fig. 2A). SS memory switched and unswitched B cells showed a similar V(D)J gene usage compared to controls. Similar to the naïve B cells from SS patients, V_H_3 was expressed more frequently (more than 50%) followed by V_H_4 and V_H_1 in both memory B cell compartments. We found a frequent usage of J_H_4 followed by J_H_6, J_H_5 and J_H_3 while J_H_1 and J_H_2 were expressed less frequently especially in the memory switched B cells. Igκ light chain usage in the two memory B cell compartments of SS patients (κ, n = 74 for memory unswitched and n = 41 for memory switched) was similar compared to HD (n = 72 for both populations) (Fig. 3A). Similarly, analysis of V_λ_ genes from memory unswitched (n = 37) and memory switched (n = 32) B cells from SS patients showed no significant difference compared to HD (n = 36 for IgM+ memory and n = 44 for IgG+ memory cells, Fig. 3B). Furthermore, no significant differences in CDR3 length and positive charges within the CDR3 were observed in both memory unswitched and switched B cells (Fig. 2B).

**Figure 4:**
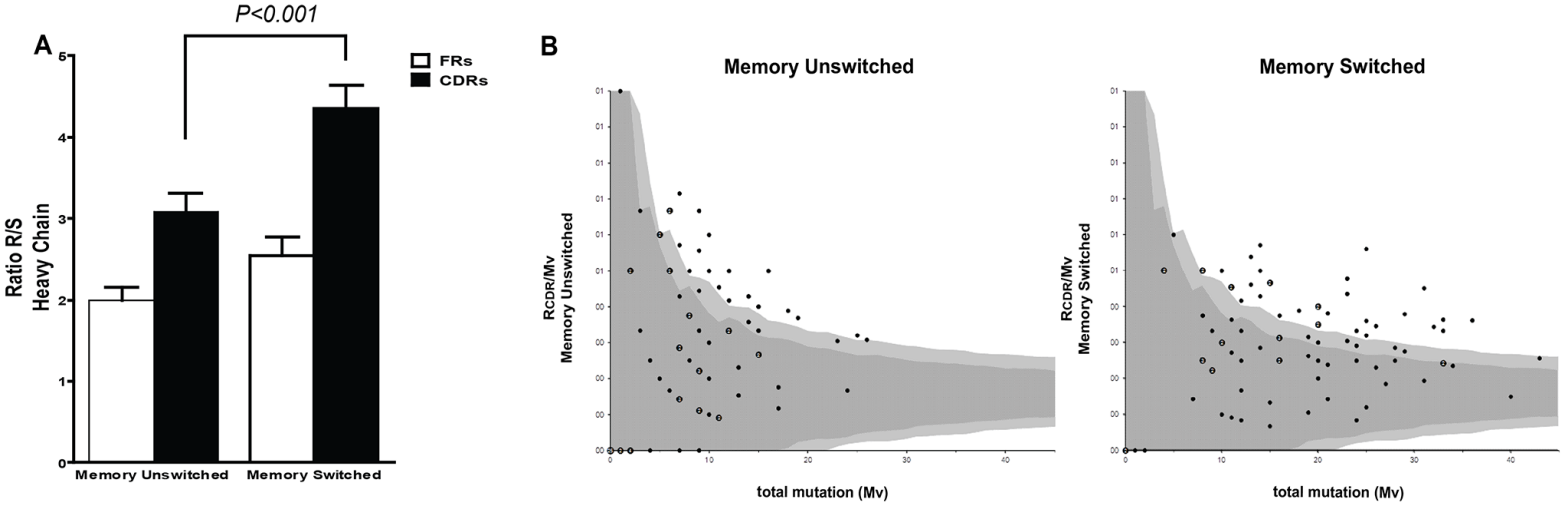
Interference of antigen selection in memory B cells from SS patients. (**A**) Replacement:silent ratio (R/S ratios) were calculated for the complementary determining regions CDRs (black) and framework regions (white) for the heavy chain of both memory unswitched and switched B cells. A significant higher R/S ratio in the CDRs was observed in memory switched vs memory unswitched B cells. (**B**) The graphs show the ratio of replacement mutations in CDR1 and CDR2 (R_CDR_) to the total number of mutations in V region (M_V_) plotted against M_V_ for the memory unswitched and switched B cells from SS patients. The dark and the light grey area indicate the 90% and 95% confidence limits for the probability of random mutations, respectively. A data point outside these areas represents a sequence that was antigen selected. The data were obtained using the Immunoglobulin Analysis Tool software [22].

Mutational analysis is normally used to study whether a B cell has experienced a positive antigen selection. Normally, non-synonymous mutations occur less frequently in the FRs in order to maintain the right Ig fold, whereas is positively selected in the CDRs to increase the antigen affinity of the Ig. Thus, the comparison of the silent (S) to replacement (R) ratios in the FR and CDR regions is used to study the presence of antigen selection. S/R ratio calculation showed a significant difference comparing CDRs and FRs of the IgH V genes in both memory unswitched and memory switched SS B cells (Fig. 4A).

Additionally, we adopted the binomial distribution method recently described by Chang and Casali [23] using the IgTA software [22] in order to calculate the probability of antigen selection based on the somatic mutation analysis in the Ig repertoire from memory unswitched and switched B cells. By this mean we estimated that 19.4% and 36.7% of the sequences from the memory unswitched and switched B cell compartment display evidence of antigen selection, respectively (Fig. 4B).

### Accumulation of autoreactive naïve B cells in the peripheral blood of SS patients suggest impairment of early tolerance checkpoints in SS

Out of all single naïve B cells isolated from the four SS patients, we managed to obtain matching IGH, IGK and IGL chains genes of 66 naïve B cells which were then cloned into specific expression vectors and used to generate rmAbs *in vitro* (SS3 = 19; SS5 = 20; SS12 = 7; SS13 = 20). As control, 45 monoclonal antibodies were expressed from IgD+ naïve B cells from HD. For full details of the repertoire and reactivity of the naïve antibodies from SS patients and HD see S2 Table and S3 Table.

**Figure 5:**
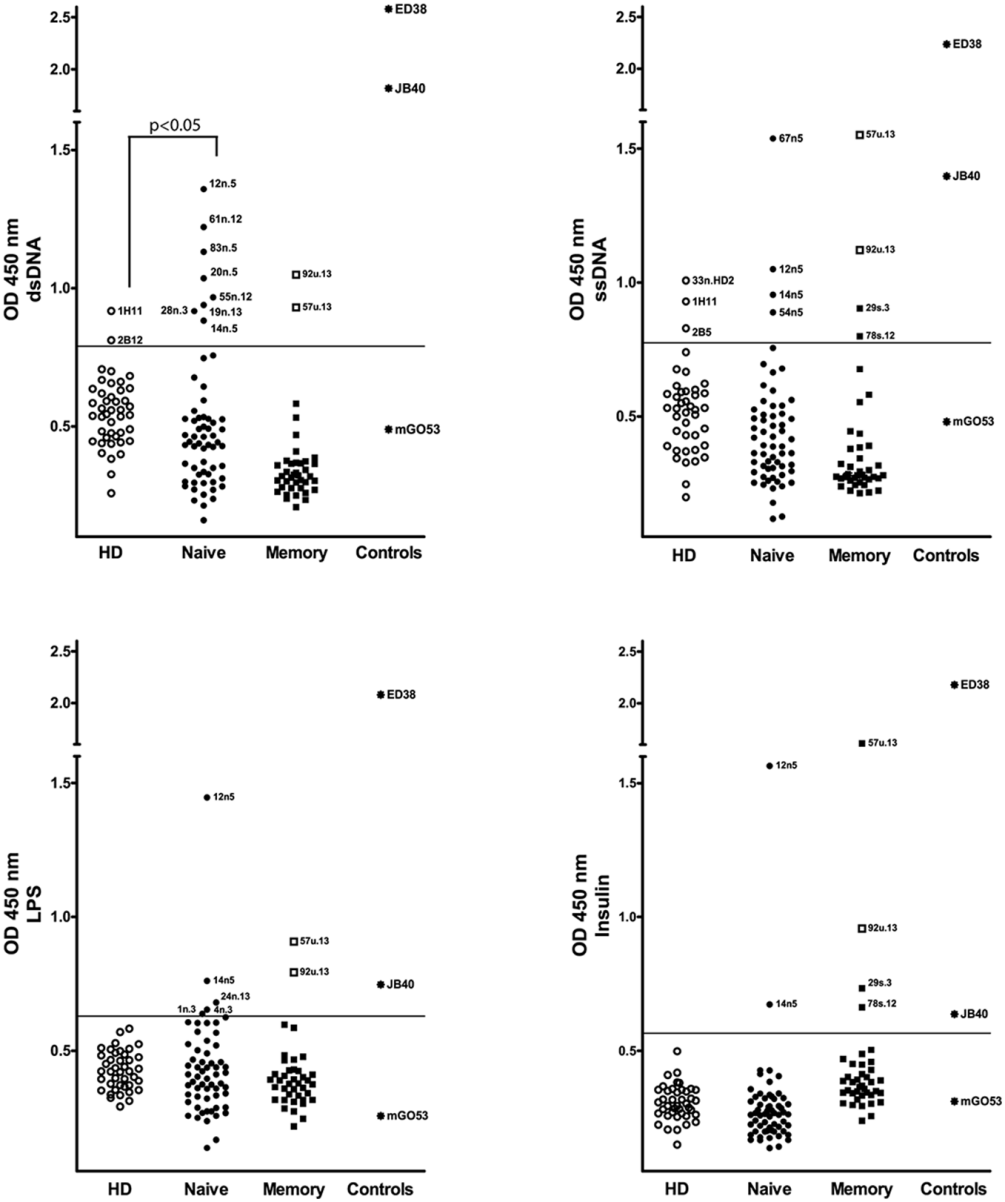
Polyreactivity of naïve and memory B cell antibodies from SS patients and HD. Naïve B cell antibodies from SS patients (n = 60), HD (n = 41) and memory B cells from SS patients (n = 39) were tested for reactivity with dsDNA (top left), ssDNA (top right), LPS (bottom left) and insulin (bottom right) by ELISA. Each graph shows the reactivity at a concentration of 1 µg/ml and it shows the result of two independent experiments. The cut-off OD (450 nm) at which antibodies were considered reactive is shown by the horizontal lines. Data points represent individual antibodies. Internal controls for polyreactivity (star dots) are shown in each graph and include mGO53 (negative; [17]), JB40 (low polyreactive; [17]), and ED38 (highly polyreactive; [17]).

**Figure 6:**
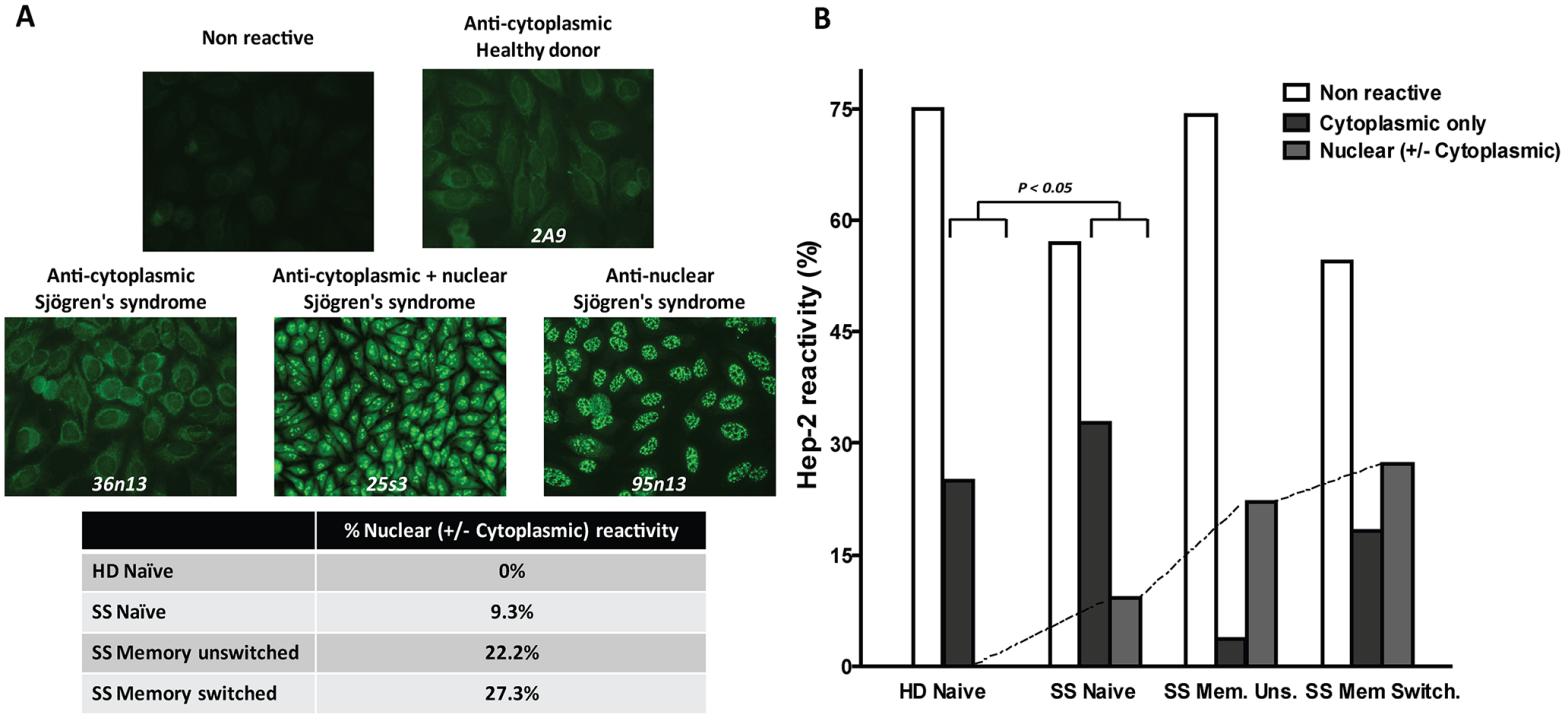
Hep-2 cell IFA self-reactivity of naïve and memory B cell antibodies from SS patients and HD. Naïve and memory B cell antibodies from SS patients and HD (naïve B cells) were tested for self-reactivity by Hep-2 cell IFA assay. (**A**) Examples of cytoplasmic, cytoplasmic and nuclear, and nuclear Hep-2 cell staining pattern are shown. (**B**) Graph bars summarize the frequency of Hep-2 cell reactive antibodies with cytoplasmic, nuclear and cytoplasmic, nuclear reactivity, and non Hep-2 cell reactive antibodies in SS patients (naïve and memory B cell antibodies) and HD (naïve B cell antibodies). *P* value compares ANA+ (cytoplasmic only and nuclear ± cytoplasmic reactive) versus ANA– (non Hep-2 reactive) naïve clones from SS patients and HD. The percentage of self-reactivity for each population is reported over each graph. The number of tested antibodies is indicated below each graph.

We used these antibodies to determine the frequency of polyreactivity and self-reactivity in the naïve B cell compartment of SS patients and controls. Antibody polyreactivity was tested using ELISAs against ssDNA, dsDNA, LPS and insulin at 1 µg/ml. As shown in Fig. 5, naïve B cell antibodies from SS patients showed very low levels of polyreactivity, which was similar to HD (3.3% vs 2.4%), as previously reported [14]. Only one antibody (SS 12n5) was found to be highly polyreactive against all four antigens (Fig. 5). Conversely, the SS naïve antibodies were more frequently reactive towards dsDNA compared to controls (13.3% versus 2.3%, respectively, p<0.05, Fig. 5), suggesting accumulation of autoreactive B cells. Autoreactive antibodies from SS naïve B cells generally displayed lower binding to dsDNA compared to highly polyreactive antibodies from memory B cells of SLE patients such as JB40 and ED38 [17]. Thus, in order to confirm the true autoreactivity of these antibodies, we next investigated the frequency of self-reactive naïve B cells from SS patients using the human epithelial cell line Hep-2 by IFA, a commonly used test for ANA in clinical diagnostic, as previously reported [14], [17]. Positive results were further classified according to the Hep-2 staining pattern as anti-nuclear, anti-cytoplasmic and mixed anti-nuclear/anti-cytoplasmic antibodies as previously reported [14]. Overall, antibodies from SS patients displayed a significantly higher prevalence of immunoreactivity to Hep-2 compared to antibodies from HD (43.1% versus 25%, p<0.05, Fig. 6B). The majority of antibodies from naïve B cells of SS patients displayed a cytoplasmic pattern while fewer antibodies showed antinuclear + cytoplasmic (8.6%) and antinuclear reactivity (1.7%, Figs. 6A and 6B). Conversely, among the 25% antibodies from HD showing positive ANA staining, none displayed a nuclear Hep-2 pattern.

Overall, these data demonstrated, both by ELISA and IFA, that autoreactive circulating naïve B cells accumulate in the peripheral blood of SS patients.

### An increased frequency of anti-nuclear autoreactivity characterises the peripheral memory unswitched and switched B cell compartment in SS patients

Out of all single memory switched and unswitched B cells isolated from SS patients, we cloned and expressed the matching IGH, IGK, and IGL chains genes from 48 individual cells (32 memory unswitched and 16 memory switched B cells). For full details of the repertoire and reactivity of the memory unswitched and switched B cell antibodies from SS patients see S4 Table and S5 Table, respectively).

We first analysed their polyreactive profile and we observed an overall frequency of polyreactivity of 7.4% in the memory unswitched B cell antibodies and 16.7% in the memory switched B cell antibodies (Fig. 5). The reported frequency of polyreactive IgM and IgG memory B cell antibodies from healthy donors is around 1% and 22%, respectively [26]. Therefore, we can conclude that the frequency of polyreactivity in the memory unswitched and switched B cell compartment is not significantly increased in SS patients compared to normal individuals as also reported in SLE patients [19].

We next analysed the frequency of self-reactive memory B cells from SS patients using the Hep-2 cells by IFA. Overall, 25.9% of the antibodies derived from IgM memory B cells displayed self-reactivity towards Hep-2 cells. The vast majority (71.4%) of these autoreactive antibodies displayed a speckled nuclear pattern with no cytoplasmic reactivity (Fig. 6). Interestingly, 50% of the nuclear-reactive unswitched memory B cells displayed germ line IgH V genes, while the remaining 50% had evidence of somatic hypermutation compatible with antigen selection. These data are extremely interesting as the reported frequency of self-reactive memory unswitched B cells in healthy individuals is only 2% [14] and further highlights that a perturbance in the circulating IgM memory B cells compartment, which bears a marginal zone-like phenotype, is likely to be critical in the development of autoimmunity in SS [7]. The analysis of the recombinant antibodies derived from memory switched B cells showed an overall frequency of self-reactivity towards Hep-2 cells of 45.5%. Among the autoreactive B cell clones, the majority (60%) displayed a strong nuclear pattern with or without cytoplasmic reactivity, again confirming an enrichment of anti-nuclear IgG memory B cells in the circulation of SS patients.

## Discussion

The presence of profound B-cell disturbances is a pivotal feature of patients with SS and has been demonstrated to play a fundamental role in disease pathogenesis and clinical evolution [27], [28]. SS patients display presence of circulating immune complexes, hypergammaglobulinemia, serum organ-specific and non-specific autoantibodies, characteristic disturbances of peripheral B cell subsets, formation of ectopic B cell follicles in the salivary glands, and increased risk of developing extranodal B cell lymphomas [27]. Furthermore, evidence that treatment with B cell depleting biological therapies is associated with clinical improvement in SS patients confirms the importance of autoreactive B cell activation in disease pathogenesis [29], [30]. However, the stage of B cell development at which the breach of self-tolerance and the onset of B cell autoreactivity develop in SS patients is still unclear.

In order to address this aspect, we single-sorted peripheral CD27−IgD+ naïve B cells, CD27+IgD+ unswitched memory B cells and CD27+IgD− switched memory B cells and generated a large number of complete (i.e., IgH + IgL V chains linked to a common IgG1 constant chain) rmAbs bearing identical specificity to the original B cells [14], [17].

Using this approach, the first novel observation that we provided is that patients with SS display a significant accumulation of autoreactive naïve B cells in the peripheral blood compared to age and sex matched healthy individuals. Although the reactivity observed appeared to be generally of low affinity, it is extremely interesting that unmutated germ line clones from SS patients display increased autoreactivity. In physiological conditions, a large proportion of self-reactive and polyreactive B cells which are normally generated in the bone marrow during B cell development (75% and 55%, respectively) are efficiently silenced at two major tolerance checkpoints before entering the peripheral compartment as naïve B cells [13], [31]. The first checkpoint removes most polyreactive B cells in the bone marrow (central tolerance checkpoint) [16], [26]. The second checkpoint in the periphery ensures that only a relatively small percentage of self-reactive and polyreactive mature naïve B cells (20% and 6%, respectively, as also confirmed in healthy donors in our study) survive [16], [26]. This physiological fraction of polyreactive and autoreactive naïve B cells is believed to form a reservoir of “natural” antibodies exerting a first-line protective role by recognizing invading pathogens [32], [33].

Thus, our evidence that an increased proportion of autoreactive CD27−IgD+ naïve B cells accumulate in the peripheral blood of SS patients strongly supports the conclusion that early B cell tolerance checkpoints are significantly impaired in SS. Similarly to other autoimmune diseases, it remains to be clarified whether this phenomenon represents a primary event in the pathogenesis of SS promoting breach of self-tolerance or is acquired in the course of chronic inflammatory stimuli which mobilize autoreactive cells from the bone marrow resulting in the peripheral accumulation of autoreactive naïve B cells. Nevertheless, our data in SS patients, together with previous reports demonstrating similar abnormalities in patients with SLE, RA and type 1 diabetes [13], [19], suggest that defects in early central/peripheral B cell tolerance checkpoints are a common denominator of autoimmune diseases.

SS patients are characterised by a distinctive distribution of peripheral B cell subpopulations. Previous analysis [27], confirmed by data from the present work, showed that pSS patients are characterised by characteristic qualitative B cell disturbances with a predominance of circulating CD27− naïve B cells and dramatically diminished peripheral CD27+ memory B cells [28], especially the circulating IgD+CD27+ memory unswitched subpopulation. Of relevance, the reduction in the peripheral memory B cell compartment seems to be related to the preferential migration of these cells into the inflamed glands, whereby they account for the majority of infiltrating B cells [28]. Therefore, we cloned and expressed rmAbs from IgD+CD27+ unswitched and IgD–CD27+ switched memory B cells. By these means we showed that around a quarter of both IgM and IgG circulating memory B cells display anti-nuclear immunoreactivity, demonstrating that with the progression of B cell maturation in SS patients there is a parallel accumulation of autoreactive memory B cells. In particular, our demonstration that circulating IgM memory B cells frequently display an autoreactive phenotype is extremely relevant as IgM memory B cells bearing a marginal zone-like phenotype infiltrate SS salivary glands and are implicated in promoting autoimmunity, chronic inflammation and evolution towards MALT lymphomas in SS patients [7]. Nevertheless, a subset of IgM memory B cells expressing high levels of CD25 have been shown to exert a regulatory role by inducing FoxP3 and CTLA4 in regulatory T cells. In this regard, further studies would be needed to ascertain whether the observed autoreactive profile of IgM memory B cells in SS patients may also represent an attempt to revert the loss of self-tolerance by inhibiting the autoreactive T cell compartment [34].

Of relevance, when tested towards the SS autoantigens Ro/SS-A and La/SS none of the non-polyreactive antibodies displayed a clear reactivity. Conversely, one of the highly polyreactive antibodies (ss12n5) reacted against both Ro/SS-A and La/SS in ELISA but also against different ENA by Western blot and was thus considered non-specific (data not shown). Overall, these results suggest that the frequency of anti-Ro/SSA and anti-La/SSB autoreactive B cells is extremely low in the peripheral compartment of SS patients possibly as a result of increased migration to the salivary glands.

Normally, in order to prevent selection of high affinity autoreactive clones a third tolerance checkpoint (the so called pre-germinal center checkpoint [26]) excludes self-reactive naïve B cells from entering B cell follicles, thereby avoiding their expansion and differentiation into memory B cells and plasma cells [26]. Thus, our data support the well-accepted notion that also later B cell tolerance checkpoints are impaired in SS patients. In particular, there is clear evidence that the mechanism of follicular exclusion is defective in SS salivary glands, allowing autoreactive naïve (and memory) B cells to enter ectopic germinal centres and undergo clonal selection and affinity maturation [8], similarly to what has been described in secondary lymphoid organs of patients with SLE [19]. Accordingly, within the inflamed salivary glands of SS, lesional memory B cells are characterised by high mutational load and evidence of ongoing clonal diversification, strongly suggesting a local antigen-driven process [35]. This is particularly evident in the context of ectopic lymphoid structures, which develop in 30–40% of SS patients [9], [11] and are characterised by the formation of functional germinal centres promoting the selection and differentiation of autoreactive B cells, leading to generation of Ro/La immunoreactive plasma cells [9], [10]. Thus, while in normal conditions, self-reactive naïve and memory B cells become anergic or produce only low affinity non-pathogenic autoantibodies [17], in SS patients peripheral autoreactive naïve and memory B cells can eventually differentiate into autoantibodies producing plasma cells, either in secondary lymphoid organs, followed by migration into the site of inflammation, or directly within ELS, and thus directly contribute to the development of autoimmunity.

Although our study was not specifically designed to assess this issue, another important observation that we provided, which confirms previous evidence in SS and other autoimmune diseases [16], [26] is that the analysis of IgH, IgK and IgL chain gene repertoire showed no significance differences in gene family usage between naïve or memory B cells from SS patients and healthy controls [19], suggesting that a skewed Ig gene family usage is not responsible for the observed accumulation of autoreactive B cells in the periphery. However, we did notice a significantly higher frequency of positively charged amino acids within the IgH CDR3 of SS naïve B cells, a feature commonly associated with autoreactive antibodies [14]. The increase of positively charged CDR3 in autoreactive naïve B cells with germ line sequences is of interest as accumulation of positively charged amino acids normally results from clonal diversification in germinal centres in secondary lymphoid organs.

## Conclusion

In summary, by analysing the immunoreactivity of single cell-derived rmAbs from naïve and memory B cells we revealed the existence of defects in both early and late B cell tolerance checkpoints in patients with Sjögren’s syndrome. In particular, i) the accumulation of circulating autoreactive naïve B cells with germ line Ig V_H_ and V_L_ genes suggest that the clearance of autoreactive B cell is already impaired at the early stages of B cell development (central and/or early peripheral tolerance checkpoints), while ii) the increased frequency of autoreactive unswitched and switched memory B cells highlights the existence of impaired pre- and/or post-germinal centre tolerance checkpoints in SS patients.

This work suggests a scenario whereby the continuous supply of newly generated autoreactive naïve B cells contributes to SS pathogenesis by promoting and maintaining the autoimmune process in secondary lymphoid organs and/or within ectopic germinal centres where impaired mechanism of follicular exclusion allows the differentiation of high affinity autoreactive memory B cells.

## Supporting Information

S1 Table **Complete list of primers used in this study.** List of primers used for Ig VH and VL gene amplification.
(DOC)

S2 Table **Ig gene repertoire analysis and reactivity of IgD+ single B cells from SS patients.** Clones highlighted in grey are those expressed as recombinant antibodies.
(XLS)

S3 Table **Ig gene repertoire analysis and reactivity of IgD+ single B cells from HD.** Clones highlighted in grey are those expressed as recombinant antibodies.
(XLS)

S4 Table **Ig gene repertoire analysis and reactivity of IgD+CD27+ single B cells from SS patients.** Clones highlighted in grey are those expressed as recombinant antibodies.
(XLS)

S5 Table **Ig gene repertoire analysis and reactivity of IgD–CD27+ single B cells from SS patients.** Clones highlighted in grey are those expressed as recombinant antibodies.
(XLS)
