# Supplementary material for: Accumulation of Self-Reactive Naïve and Memory B Cell Reveals Sequential Defects in B Cell Tolerance Checkpoints in Sjögren’s Syndrome
Source: PLoS One. 2014 Dec 23;9(12):e114575. doi: 10.1371/journal.pone.0114575 (PMC4275206; doi:10.1371/journal.pone.0114575)
Supplement: Supplementary file 1 [file pone.0114575.s001.doc]

1st PCR HC	Forward 5' to 3'
5' L-VH 1	ACAGGTGCCCACTCCCAGGTGCAG
5' L-VH 3	AAGGTGTCCAGTGTGARGTGCAG
5' L-VH 4/6	CCCAGATGGGTCCTGTCCCAGGTGCAG
5' L-VH 5	CAAGGAGTCTGTTCCGAGGTGCAG
	Reverse 3' to 5'
3' Cμ CH1 ext	GGAAGGAAGTCCTGTGCGAGGC
3' CγCH1 ext	GGAAGGTGTGCACGCCGCTGGTC
3' Cα CH1 ext	TGGGAAGTTTCTGGCGGTCACG
2nd PCR HC	Forward 5' to 3'
5' AgeI VH1	CTGCAACCGGTGTACATTCCCAGGTGCAGCTGGTGCAG
5' AgeI VH1/5	CTGCAACCGGTGTACATTCCGAGGTGCAGCTGGTGCAG
5' AgeI VH 1-18	CTGCAACCGGTGTACATTCCCAGGTTCAGCTGGTGCAG
5' AgeI VH 1-24	CTGCAACCGGTGTACATTCCCAGGTCCAGCTGGTACAG
5' AgeI VH3	CTGCAACCGGTGTACATTCTGAGGTGCAGCTGGTGGAG
5' AgeI VH3-23	CTGCAACCGGTGTACATTCTGAGGTGCAGCTGTTGGAG
5' AgeI VH3-33	CTGCAACCGGTGTACATTCTCAGGTGCAGCTGGTGGAG
5' AgeI VH 3-9	CTGCAACCGGTGTACATTCTGAAGTGCAGCTGGTGGAG
5' AgeI VH4	CTGCAACCGGTGTACATTCCCAGGTGCAGCTGCAGGAG
5' AgeI VH 4-34	CTGCAACCGGTGTACATTCCCAGGTGCAGCTACAGCAGTG
5' AgeI VH4-39	CTGCAACCGGTGTACATTCCCAGCTGCAGCTGCAGGAG
5' AgeI VH 6-1	CTGCAACCGGTGTACATTCCCAGGTACAGCTGCAGCAG
	Reverse 3' to 5'
3' Cμ CH1 int	GGGAATTCTCACAGGAGACGA
3' CγIgG int	GTTCGGGGAAGTAGTCCTTGAC
3' Cα CH1-2 int	GTCCGCTTTCGCTCCAGGTCACACT
1st PCR λ LC	Forward 5' to 3'
5' L Vλ 1	GGTCCTGGGCCCAGTCTGTGCTG
5' L Vλ 2	GGTCCTGGGCCCAGTCTGCCCTG
5' L Vλ 3	GCTCTGTGACCTCCTATGAGCTG
5' L Vλ 4/5	GGTCTCTCTCSCAGCYTGTGCTG
5' L Vλ 6	GTTCTTGGGCCAATTTTATGCTG
5' L Vλ 7	GGTCCAATTCYCAGGCTGTGGTG
5' L Vλ 8	GAGTGGATTCTCAGACTGTGGTG
	Reverse 3' to 5'
3' Cλ	CACCAGTGTGGCCTTGTTGGCTTG
2nd PCR l LC	Forward 5' to 3'
5' AgeI Vλ 1	CTGCTACCGGTTCCTGGGCCCAGTCTGTGCTGACKCAG
5' AgeI Vλ 2	CTGCTACCGGTTCCTGGGCCCAGTCTGCCCTGACTCAG
5' AgeI Vλ 3	CTGCTACCGGTTCTGTGACCTCCTATGAGCTGACWCAG
5' AgeI Vλ 4/5	CTGCTACCGGTTCTCTCTCSCAGCYTGTGCTGACTCA
5' AgeI Vλ 6	CTGCTACCGGTTCTTGGGCCAATTTTATGCTGACTCAG
5' AgeI Vλ 7/8	CTGCTACCGGTTCCAATTCYCAGRCTGTGGTGACYCAG
	Reverse 3' to 5'
3' XhoI Cλ	CTCCTCACTCGAGGGYGGGAACAGAGTG
1st PCR κ LC	Forward 5' to 3'
5' L Vκ 1/2	ATGAGGSTCCCYGCTCAGCTGCTGG
5' L Vκ 3	CTCTTCCTCCTGCTACTCTGGCTCCCAG
5' L Vκ 4	ATTTCTCTGTTGCTCTGGATCTCTG
	Reverse 3' to 5'
3' Cκ 543	GTTTCTCGTAGTCTGCTTTGCTCA
2nd PCR k LC	Forward 5' to 3'
5' Pan Vκ	ATGACCCAGWCTCCABYCWCCCTG
	Reverse 3' to 5'
3' Cκ 494	GTGCTGTCCTTGCTGTCCTGCT
Specific k LC	Forward 5' to 3'
5' AgeI Vκ 1-5	CTGCAACCGGTGTACATTCTGACATCCAGATGACCCAGTC
5' AgeI Vκ 1-9	TTGTGCTGCAACCGGTGTACATTCAGACATCCAGTTGACCCAGTCT
5' AgeI Vκ 1D-43	CTGCAACCGGTGTACATTGTGCCATCCGGATGACCCAGTC
5' AgeI Vκ 2-24	CTGCAACCGGTGTACATGGGGATATTGTGATGACCCAGAC
5' AgeI Vκ 2-28	CTGCAACCGGTGTACATGGGGATATTGTGATGACTCAGTC
5' AgeI Vκ 2-30	CTGCAACCGGTGTACATGGGGATGTTGTGATGACTCAGTC
5' Age Vκ 3-11	TTGTGCTGCAACCGGTGTACATTCAGAAATTGTGTTGACACAGTC
5' Age Vκ 3-15	CTGCAACCGGTGTACATTCAGAAATAGTGATGACGCAGTC
5' Age Vκ 3-20	TTGTGCTGCAACCGGTGTACATTCAGAAATTGTGTTGACGCAGTCT
5' Age Vκ 4-1	CTGCAACCGGTGTACATTCGGACATCGTGATGACCCAGTC
	Reverse 3' to 5'
3' BsiWI Jκ 1/4	GCCACCGTACGTTTGATYTCCACCTTGGTC
3' BsiWI Jκ 2	GCCACCGTACGTTTGATCTCCAGCTTGGTC
3' BsiWI Jκ 3	GCCACCGTACGTTTGATATCCACTTTGGTC
3' BsiWI Jκ 5	GCCACCGTACGTTTAATCTCCAGTCGTGTC
